# Predicting peripartum blood transfusion: focusing on pre-pregnancy characteristics

**DOI:** 10.1186/s12884-019-2646-3

**Published:** 2019-12-05

**Authors:** Yung-Taek Ouh, Kyu-Min Lee, Ki Hoon Ahn, Soon-Cheol Hong, Min-Jeong Oh, Hai-Joong Kim, Sung Won Han, Geum Joon Cho

**Affiliations:** 1 Department of Obstetrics and Gynecology, Korea University College of Medicine, Seoul, Korea; 2 School of Industrial Management Engineering, Korea University, 145 Anam-Ro, Seongbuk-Gu, Seoul, 02841 Republic of Korea

**Keywords:** Peripartum, Blood transfusion, Postpartum hemorrhage

## Abstract

**Background:**

Obstetric hemorrhage is one of the most common causes of obstetrical morbidity and mortality, and transfusion is the most important management for hemorrhage. The aim of our study was to investigate the pre-pregnancy and pregnancy risk factors for peripartum transfusion.

**Methods:**

Women who delivered a baby from 2010 to 2014 in Korea and participated in the Korean National Health Screening Program for Infants and Children were included. To analyze pre-pregnant risk factors for peripartum transfusion, an additional analysis was done for women who underwent a National Health Screening Examination within 1 year before pregnancy, including maternal waist circumference, body mass index, blood pressure, laboratory tests and history of smoking. Multivariable logistic regression analysis was used to estimate the risk factors for peripartum transfusion.

**Results:**

Of the total 1,980,126 women who met the inclusion criteria, 36,868 (1.86%) were transfused at peripartum. In a multivariable regression model, the pregnancy risk factors for peripartum transfusion included maternal age above 35 years [odds ratio (OR): 1.41; 95% confidence interval (CI): 1.32–1.50], preterm birth (OR: 2.39; 95% CI: 2.15–2.65), and maternal hypertension (OR: 2.49; 95% CI: 2.24–2.77). Pre-pregnancy risk factors including fasting glucose level of more than 126 mg/dL (OR: 1.11; 95% CI: 1.02–1.20), current-smoker status (OR: 1.20; 95% CI: 1.06–1.37), and waist-circumference less than 80 cm (OR: 1.18; 95% CI: 1.06–1.30) were independently associated with peripartum blood transfusion.

**Conclusions:**

Several pre-pregnancy and pregnancy risk factors were associated with peripartum blood transfusion. Some identified factors are modifiable before conception, and our study validated peripartum blood transfusion as a form of triage.

## Background

Obstetric hemorrhage is a life-threatening problem and a major cause of maternal morbidity and mortality, with an incidence that has increased recently in developed countries [1, 2]. Blood transfusion is one of the most important management methods for this condition, especially in critical status patients. During hypovolemic status caused by obstetric hemorrhage, transfused blood sustains circulating blood volume, oxygenates organ tissues and prevents disseminated intravascular coagulopathy. Transfusion during delivery has increased worldwide, driven by increases in uterine atony, cesarean deliveries, and multifetal pregnancies [3]. A reduction in the transfusion threshold and improvement of accessibility are also related to this increase [4].

Both vaginal delivery and cesarean section procedures are often accompanied by relatively larger amounts of hemorrhage, which can be resolved by volume replacement. Although immediate blood transfusion should be implemented and could save lives in cases of postpartum hemorrhage (PPH), it has been known to result in adverse effects, including infection, allergic reactions, posterior reversible encephalopathy syndrome, lung injury, and thromboembolism [5, 6]. For these potential adverse effects of transfusion, it is necessary to triage the pregnant women who are at high risk of peripartum transfusion to reduce obstetrical morbidity and mortality.

It has been difficult to predict and cope with peripartum transfusion because key differences exist in maternal, antepartum, and postpartum characteristics, with changes occurring every moment during labor. An accurate method to predict which pregnant women are at high risk for peripartum transfusion is needed to improve pregnancy-associated complication management and optimize health care institute resource allocation. Therefore, we analyzed a large and nationally representative dataset in Korea to provide new insights into the risk of peripartum transfusion. We aimed to analyze the pre-pregnancy and pregnancy risk factors for peripartum transfusion.

## Methods

In Korea, 97% of the population is enrolled in the Korea National Health Insurance (KNHI) program. All claims information for these individuals is contained within the KNHI claims database. For this study, we used the KNHI claims database, which due to its comprehensive nature contains nearly all information about the prevalence rates of different diseases and procedures in Korea, with the exception of procedures not covered by insurance such as cosmetic surgery. The KNHI service provides a biannual National Health Screening Examination (NHSE) program for adults. The NHSE consists of a health interview and physical examination. Similarly, the KNHI service provides a National Health Screening Program for Infants and Children (NHSP-IC), which was introduced in 2007 and includes seven consecutive health examinations based on age groups (4–9 months, 9–18 months, 18–30 months, 30–42 months, 42–54 months, 54–66 months, and 66–80 months). Data from this program, including physical examination, anthropometric examination, and developmental screening findings, are also contained within the program’s database. This study was thus conducted by merging the KNHI claims database, NHSE data, and NHSP-IC data.

This study was approved by the Institutional Review Board of the Korea University Medical Center. Anonymized and deidentified information for participants was used for analysis, so the requirement for informed consent or parental permission was waived.

### Dataset and outcomes

**Fig. 1:**
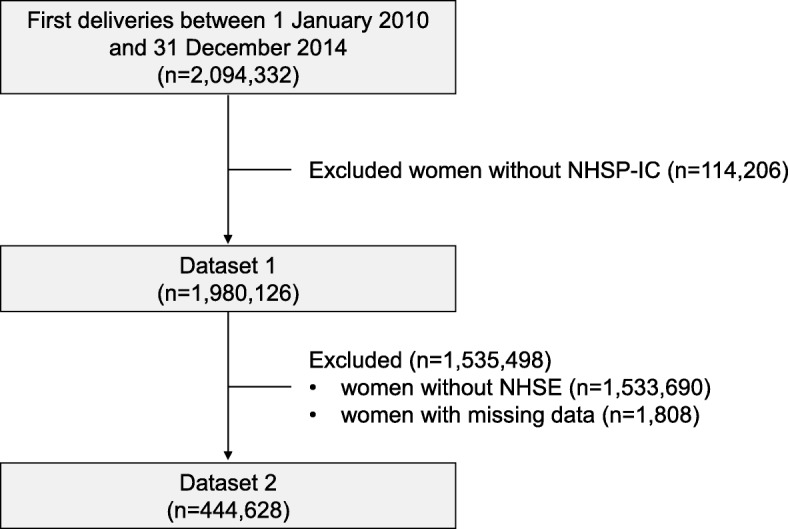
Flowchart of participant enrollment. NHSE: National Health Screening Examination; NHSP-IC: National Health Screening Program for Infants and Children

Figure 1 presents a flowchart of study participants’ enrollment. To evaluate the pregnancy risk factors for peripartum transfusion, using KNHI claims data, we identified all nulliparous women who had given birth between January 1, 2010 and December 31, 2014. Women were excluded from analysis if their offspring had not undergone at least one of the seven consecutive NHSP-IC examinations (dataset 1). For the pre-pregnancy risk factor outcomes, we merged dataset 1 and NHSE data. Women were included in the analysis only if they underwent an NHSE within 1 year prior to their pregnancy (dataset 2). Using the KNHI claims database, we obtained information on blood transfusions in the peripartum period.

### Variables

Variables for pregnancy factors were extracted from dataset 1. Using the KNHI claims dataset, age, PPH, placental abruption, placenta previa, and preeclampsia were identified according to International Classification of Diseases, 10th revision codes. Based on the KNHI claims dataset, information on the presence of multiple pregnancies, the delivery mode, UAE, and hysterectomy was gathered. Using NHSP-IC data, preterm birth, birthweight, and neonatal sex were also identified. Preterm birth was defined as a gestational age < 37 weeks [7]. Low birth weight (LBW) and large size for gestational age (LGA) were defined as birth weight < 2.5 kg and > 4.0 kg, respectively [8].

Pre-pregnancy factors were evaluated using the NHSE data. The health examination included the calculation of body mass index (BMI, in kg/m^2^) using height and weight measurements. Obesity was defined as a BMI of 25 kg/m^2^ or more [9]. Waist circumference (WC) was measured at the narrowest point between the lower border of the rib cage and the iliac crest during minimal respiration with a cutoff level of 80 cm [10]. Blood pressure (BP) was measured using a standard mercury sphygmomanometer. Hypertension (HTN) was defined as a systolic/diastolic BP ≥ 130/85 mmHg or the current use of antihypertensive medication. Blood samples were obtained after a fast of at least 8 h. The levels of fasting glucose, aspartate aminotransferase (AST), alanine aminotransferase (ALT), and total cholesterol (TC) were measured. Diabetes mellitus (DM) was defined as a fasting glucose ≥126 mg/dL or the current use of antidiabetic medication. Abnormal liver function test (LFT) was defined as the finding of an AST ≥ 30 mg/dL or ALT ≥30 mg/dL [11]. High TC level was defined as TC ≥ 200 mg/dL [12]. Smoking status was identified using health questionnaires.

### Statistical analysis

Continuous and categorical variables were expressed as means ± standard deviations and percentages, respectively. Clinical characteristics were compared using the t-test for continuous variables and the chi-squared test for categorical variables. Multivariable logistic regression analysis was used to estimate the adjusted odds ratio (OR) and the 95% confidence intervals (CIs). All tests were two-sided, and *p* <  0.05 was considered to be statistically significant. Statistical analyses were performed using SAS for Windows, version 9.4 (SAS Inc., Cary, NC, USA).

## Results

Of the 2,094,332 deliveries recorded in the database, 114,206 women did not have NHSP-IC data. Among a total of 1,980,126 women who met our inclusion criteria, 36,868 women were transfused a week before birth until 1 month after birth and 1,943,258 women were not. Maternal, obstetric, and postpartum characteristics of PPH requiring transfusion cases are presented in Table 1. The rates of peripartum blood transfusion were associated with maternal age. In addition, the incidence of peripartum blood transfusion was higher in women with multifetal pregnancy, nulliparity, cesarean deliveries, and preterm deliveries before 37 weeks. Furthermore obviously, women diagnosed with maternal HTN, placental abruption, placenta previa, uterine embolization, and hysterectomy had higher rates of peripartum blood transfusion.

**Table 1:** Distribution of peripartum variables and differences associated with peripartum blood transfusion

	No transfusion 1,943,258 (98.14%)	Transfusion 36,868 (1.86%)	*p*-value
Maternal age (years) mean ± SD	31.16 ± 3.86	32.14 ± 4.26	< 0.001
Maternal age ≥ 35 years, N (%)	349,119 (17.97%)	10,177 (27.60%)	< 0.001
Multifetal pregnancy, N (%)	27,331 (1.41%)	2761 (7.49%)	< 0.001
Nulliparity, N (%)	927,079 (47.74%)	15,968 (43.31%)	< 0.001
Cesarean delivery, N (%)	713,992 (36.74%)	21,388 (58.01%)	< 0.001
Gestational age at births < 37 weeks, N (%)	56,826 (2.92%)	5243 (14.22%)	< 0.001
Neonatal weight (kg) mean ± SD	3.19 ± 0.47	3.03 ± 0.67	< 0.001
2.5–4.0 kg, N (%)	1,793,764 (92.31%)	29,503 (80.02%)	< 0.001
< 2.5 kg, N (%)	80,842 (4.16%)	5743 (15.58%)	
≥ 4.0 kg, N (%)	68,652 (3.53%)	1622 (4.40%)	
Neonatal sex (male), N (%)	996,211 (51.26%)	18,111 (49.12%)	< 0.001
Hypertension, N (%)	35,335 (1.82%)	2561 (6.95%)	< 0.001
Postpartum hemorrhage, N (%)	150,259 (7.73%)	16,072 (43.59%)	< 0.001
Placental abruption, N (%)	5925 (0.30%)	1033 (2.80%)	< 0.001
Placenta previa, N (%)	17,391 (0.89%)	5479 (14.86%)	< 0.001
Embolization, N (%)	151 (0.01%)	2450 (6.65%)	< 0.001
Hysterectomy, N (%)	229 (0.01%)	1569 (4.26%)	< 0.001

Of the 444,628 women who had an NHSE within 1 year before conception, 7960 women were transfused a week before birth until 1 month after birth. Table 2 shows the distribution of variables including maternal characteristics before conception. Peripartum blood transfusion rates were higher in women who had higher BPs before conception as well as lower hemoglobin, higher fasting glucose, and higher liver enzyme levels. In addition, women who were smokers before conception had higher rates of peripartum blood transfusion.

**Table 2:** Distribution of pre-pregnancy variables and differences associated with peripartum blood transfusion in women undergoing NHSE within one year before conception

	No Transfusion 436,668 (98.21%)	Transfusion 7960 (1.79%)	*p*-value
Waist circumference (cm) ≥ 80	47,317 (10.80%)	838 (10.49%)	0.3695
Body mass index (kg/m^2^)	21.05 ± 2.87	21.09 ± 2.93	0.2
Body mass index (kg/m^2^) ≥ 25	39,454 (9.00%)	750 (9.38%)	0.2428
Systolic blood pressure (mmHg)	110.1 ± 10.84	110.5 ± 11.24	< 0.001
Diastolic blood pressure (mmHg)	69.19 ± 8.12	69.55 ± 8.35	< 0.001
Blood pressure ≥ 130/85 (mmHg)	30,349 (6.92%)	664 (8.30%)	< 0.001
Hemoglobin	12.93 ± 1.01	12.70 ± 1.19	< 0.001
Fasting glucose (mg/dL)	87.81 ± 11.36	88.58 ± 12.54	< 0.001
Fasting glucose ≥126	42,380 (9.67%)	900 (11.26%)	< 0.001
Total cholesterol (mg/dL)	176.9 ± 31.55	177.2 ± 36.46	0.4163
Total cholesterol (mg/dL) ≥ 200	87,585 (19.98%)	1646 (20.59%)	0.1772
AST (IU/L)	19.24 ± 12.77	19.48 ± 11.03	< 0.05
AST (IU/L) ≥ 30	18,724 (4.27%)	385 (4.81%)	< 0.05
ALT (IU/L)	15.38 ± 16.83	15.70 ± 16.73	0.087
ALT (IU/L) ≥ 30	21,314 (4.86%)	444 (5.55%)	< 0.01
Smoking			
Never	407,408 (93.21%)	7403 (92.89%)	< 0.05
Ever	15,480 (3.54%)	264 (3.31%)	
Current	14,190 (3.25%)	303 (3.80%)	

*Abbreviations*: *AST* Aspartate aminotransferase, *ALT* Alanine aminotransferase

Table 3 shows adjusted multivariate regression models for the pregnancy risk of peripartum blood transfusion. Among pregnancy variables, maternal age ≥ 35 years (OR: 1.43; 95% CI: 1.40–1.47), multifetal pregnancy (OR: 2.26; 95% CI: 2.15–2.39), nulliparity (OR: 1.19; 95% CI: 1.16–1.22), and cesarean delivery (OR: 1.64; 95% CI: 1.60–1.68) were significantly associated with the risk of peripartum blood transfusion. Additionally, gestational age < 37 weeks (OR: 2.53; 95% CI: 2.41–2.65), neonatal weight under 2.5 kg (OR: 1.64; 95% CI: 1.56–1.72) or above 4.0 kg (OR: 1.40; 95% CI: 1.33–1.48), and maternal HTN during pregnancy (OR: 2.41; 95% CI: 2.29–2.53) were independently associated with peripartum blood transfusion.

**Table 3:** Adjusted multivariable logistic regression models for peripartum blood transfusion associated with pregnancy factors

	Odds ratio	95% CI
Maternal age ≥ 35 years	1.43	1.40–1.47
Multifetal pregnancy	2.26	2.15–2.39
Nulliparity	1.19	1.16–1.22
Cesarean delivery	1.64	1.60–1.68
Gestational age at births < 37 weeks	2.53	2.41–2.65
Neonatal weight (kg)		
< 2.5 kg	1.64	1.56–1.72
≥ 4.0 kg	1.40	1.33–1.48
Neonatal sex (male)	0.95	0.93–0.97
Hypertension	2.41	2.29–2.53
Postpartum hemorrhage	9.09	8.87–9.31
Placental abruption	4.74	4.37–5.14
Placenta previa	13.65	13.12–14.20
Embolization	218.90	184.34–259.95
Hysterectomy	105.11	89.81–123.01

*Abbreviations*: *CI* Confidence interval

Separately in Table 4, the independent pre-pregnancy risk factors associated with peripartum blood transfusion were lower hemoglobin (OR: 0.81; 95% CI: 0.79–0.82), fasting glucose above 126 mg/dL (OR: 1.15; 95% CI: 1.08–1.24), and current-smoker (OR: 1.24; 95% CI: 1.11–1.40) compared with never-smoker. Interestingly, women with a preconception WC of 80 cm or less had an increased risk of PPH (OR: 1.12; 95% CI: 1.03–1.23).

**Table 4:** Adjusted multivariable logistic regression models for peripartum blood transfusion associated with pre-pregnancy factors

	Odds ratio	95% CI
Waist-circumference (cm) < 80	1.12	1.03–1.23
Body mass index (kg/m^2^) ≥ 25	1.06	0.97–1.17
Blood pressure ≥ 130/85 (mmHg)	1.25	1.15–1.35
Hemoglobin	0.81	0.79–0.82
Fasting glucose ≥126	1.15	1.08–1.24
Total cholesterol (mg/dL) ≥ 200	1.06	0.99–1.11
AST (IU/L) ≥ 30	1.05	0.93–1.20
ALT (IU/L) ≥ 30	1.14	1.01–1.28
Smoking		
Never	1	.
Ever	0.97	0.85–1.09
Current	1.24	1.11–1.40

*Abbreviations*: *CI* Confidence interval, *AST* Aspartate aminotransferase, *ALT* Alanine aminotransferase

## Discussion

In the present research, we evaluated the risk factors of peripartum transfusion in pregnant women and found that maternal age, multiple pregnancies, fetal sex, cesarean section, preterm delivery, and preeclampsia were associated with an increased risk of peripartum transfusion, findings which are consistent with results from previous studies [4, 13, 14]. Women with abnormal placentation, such as abruption and previa, had an increased risk of postpartum transfusion. Neonatal weight, LBW, and LGA were also associated with peripartum transfusion. In addition, pre-pregnancy factors, which included WC, low hemoglobin level, fasting glucose, and current smoking habit had an association with peripartum transfusion.

PPH encompasses several related predisposing factors for peripartum transfusion. Because the diagnosis of PPH is subjective, it could be substituted for peripartum transfusion. PPH is one of the most common causes of obstetrical morbidity and mortality [15]. It accounts for 30% or more of all maternal deaths, especially in Asia [16]. It is an obstetric emergency and physicians including anesthesiologists and intensivists are primarily responsible for hemodynamic management [17]. Recently, PPH rates in developed countries have been increasing, especially in a manner attributable to uterine atony [1, 2, 18, 19]. The causes of PPH were uterine atony, abnormal placentation, genital tract trauma, and coagulopathy [20]. Although several risk factors for PPH have been widely established, it also often occurs with no identifiable obstetrical risk factors and is not preventable. The most important risk factor for PPH is probably an overdistended uterus, which accounts for 90% of all PPHs [21]. Because the average blood flow rate to the uterus during labor is 600 mL per minute, the lack of uterine contractions can cause severe PPH requiring transfusion, hypovolemic shock, and even death [22].

Although we were not able to identify preeclampsia, maternal HTN during pregnancy was observed to be an independent risk factor for the development of PPH, as noted in previous studies [22–24]. Compared to normal pregnancy, preeclampsia is characterized by systemic vascular resistance, lower cardiac output, and hypovolemia [25]. Dehydrated pregnant women are vulnerable to hemodynamic instability caused by PPH. An imbalance between angiogenic and antiangiogenic factors in the maternal blood is associated with gestational HTN [26]. In addition, deficient platelet count and HTN aggravated blood loss and required transfusion [27]. Preeclampsia is associated with placental ischemia, which consequently reduces the placental growth factor (PIGF) level, with increased coagulopathy resulting from activation of the fibrinolytic system, platelet activation, and a decrease in platelet count. PPH is defined as a maternal serum PIGF level < 122 pg/mL at 22 to 24 weeks of gestation [28].

Abnormal neonatal weight, both high and low, is one of the variables that had an impact on peripartum transfusion. The finding that high birth weight was associated with such may suggest the presence of atony due to an overdistended uterus that has lost the ability to contract and so the risk of substantial blood loss is increased [29]. This is the same mechanism that drives the increased risk of transfusion in multifetal pregnancy [30]. On the contrary, low birth weight does not lead to uterine atony. One possible reason may be complications that can occur in pregnancy including preterm delivery, preeclampsia, and placental abruption [31].

Our results suggest that a sex difference existed in the risk for peripartum transfusion, which was higher when the fetus was female. Although fetal sex has a significant effect on pregnancy outcome and complications [32], conclusions on the association between fetal sex and pregnancy outcomes remain controversial. To date, pathophysiologic evidence for sex differences is largely unknown. Our results are consistent with previous research, in that female fetuses are associated with an increased incidence of PPH, malpresentation, and FGR [33]. However, placental origins rather than fetal origins were related to the different outcomes. Female fetuses have larger placentas relative to their birth weight compared to male fetuses [34]. Pregnancies with a female fetus were also prone to complications due to excessive placental invasion [35]; more peripartum transfusion occurred with female than male fetuses. Conversely, male fetuses showed an increased risk for many adverse perinatal complications such as gestational DM, perinatal mortality, fetal macrosomia, placental abruption, and placenta previa [36–39]. Male fetus placentas were also more likely to have reversed end-diastolic umbilical artery flow than female fetus placentas [34]. Importantly, the heterogeneity of these results may be due to different populations, so a worldwide study should be conducted.

An important strength of our study is its comprehensive dataset following conception; data from the NHSE taken before conception with peripartum transfusion is considerably important. Severe postpartum anemia was strongly associated with predelivery hemoglobin level in a previous report [40]. However, there have not been any studies about the risk of preconception anemia for PPH. Our results showed that preconception hemoglobin was associated with postpartum transfusion, and this result is clinically relevant because preconception anemia is a modifiable risk factor. In addition, hemoglobin levels in women who planned to become pregnant were important, because anemia affects 15 to 30% of antenatal women and is associated with maternal morbidity [41, 42].

In a recent cohort study, women with increased pre-pregnancy WC were at risk of adverse pregnancy outcomes including gestational DM, primary cesarean section, and LGA [43]. A number of studies have found obesity to be closely associated with PPH [44, 45]. Contrary to previous studies, the results of this study suggested that central obesity before conception was associated with a decreased risk of peripartum transfusion. In general, obese women had a higher intake of iron than underweight women [46]. In addition, the positive association between WC and serum ferritin was reported in a previous study [47]. On the other hand, our results showed that maternal BMI before conception was not associated with peripartum transfusion, which was consistent with prior research [48]. In addition to PPH, length of labor, third-degree tear, low Apgar score, and shoulder dystocia were not different according to BMI [48].

Women with current-smoker status within 1 year before conception had an increased risk of peripartum transfusion, while women who had quit smoking by the time of their NHSE did not. This may related to placental abruption, which was a significant cause of PPH [49–51]. In addition, the use of tobacco increased the risk of placenta previa, preterm birth, intrauterine growth restriction, and fetal sudden death [52]. Quitting smoking before conception seems to reduce the risk of abruption and placenta previa compared to mothers who continued to smoke [53]. Unfortunately, our data did not include maternal smoking status at conception or antepartum period, so further studies are needed.

The blood transfusion rate in our report was 1.9%, which was slightly higher than in previous studies that reported a < 1% rate [2, 54]. This may be because we included all transfused cases a week before birth until 1 month after birth, which is an extended period of time. We also used a different strategy for blood transfusion compared to that used in previous studies. Blood transfusion is the most effective and essential management option against severe hemorrhage [55]. Nevertheless, the risks of blood transfusion must be considered in managing PPH, although blood transfusions are lifesaving in most severe cases. Previous observational studies showed that blood transfusion in the critically ill may have a deleterious effect on clinical outcomes, independent of illness severity or hemoglobin level [56, 57]. Blood transfusion may induce not only circulatory overload, acute lung injury, and allergic reaction but also thromboembolism and stroke [5]. Peripartum transfusion increased the incidence of stroke more than 10-fold, although women who needed transfusions may also be at high risk for other stroke factors such as preeclampsia and PPH [58]. A rapid increase in hemoglobin and hematocrit induced enhanced blood viscosity, possibly increasing the risk of thrombosis [59]. Furthermore, rare neurologic complications such as angiopathy and encephalopathy have been reported after blood transfusion, that result from hypertensive encephalopathy [60]. In addition, intraoperative transfusion was found to enhance inflammatory responses and consequently increase postoperative morbidity in cardiac surgery, in which neutrophil activation, interleukin-6, and C-reactive protein are involved [61].

Readers should be aware of the limitations in the present study. Our database was based on the NHSP-IC in Korea, which contains large amounts of population-based information. We established our primary endpoint as transfusion to predict PPH; however, criteria for the management of PPH depend on local transfusion policies. Although there was an alternative definition of PPH as a drop in hemoglobin level, which was considered to be the most objective option [26], assessments of hemoglobin change were not available for all women. Nevertheless, our data will be useful for women who are currently pregnant and who have risk factors for peripartum transfusion, as our results are based on the largest sample size reported to date.

## Conclusions

Our study offers a benefit for clinicians predicting and screening women at higher risk of peripartum transfusion, especially as it focuses not only on intrapartum but also pre-pregnancy risk factors. Although many of the identified variables cannot be prevented, pregnant women with these risk factors can be managed ahead of delivery as well as closely monitored. Given the limited ability to screen for PPH, physicians need to focus on developing and enforcing strategies for predicting high risk for peripartum transfusion. Women who are at higher risk for peripartum transfusion should be identified, and the risk of transfusion itself should be explained. Furthermore, if a hospital does not have prompt blood transfusion supplies available, it is necessary to consult with the patient about transfer to a tertiary hospital.

## Data Availability

The data that support the findings of this study are available from the National Health Insurance Service (NHIS), but restrictions apply to the availability of these data, which were used under license for the current study and so are not publicly available. Data are however available from the authors upon reasonable request and with permission of the NHIS. The results do not necessarily represent the opinion of the National Health Insurance Corporation.

## Abbreviations

ALT: Alanine aminotransferase
AST: Aspartate aminotransferase
BMI: Body mass index
BP: Blood pressure
CIs: Confidence intervals
DM: Diabetes mellitus
HTN: Hypertension
KNHI: Korea National Health Insurance
LBW: Low birth weight
LFT: Liver function test
LGA: Large size for gestational age
NHSE: National Health Screening Examination
NHSP-IC: National Health Screening Program for Infants and Children
OR: Odds ratio
PPH: Postpartum hemorrhage
TC: Total cholesterol
WC: Waist circumference
